# Landscape use and co-occurrence pattern of snow leopard (*Panthera uncia*) and its prey species in the fragile ecosystem of Spiti Valley, Himachal Pradesh

**DOI:** 10.1371/journal.pone.0271556

**Published:** 2022-07-21

**Authors:** Amira Sharief, Vineet Kumar, Hemant Singh, Tanoy Mukherjee, Ritam Dutta, Bheem Dutt Joshi, Saurav Bhattacharjee, Chinnasamy Ramesh, Kailash Chandra, Mukesh Thakur, Lalit Kumar Sharma

**Affiliations:** 1 Zoological Survey of India, New Alipore, Kolkata, West Bengal, India; 2 Wildlife Institute of India, Dehradun, Uttarakhand, India; MARE – Marine and Environmental Sciences Centre, PORTUGAL

## Abstract

The snow leopard (*Panthera uncia*) plays a vital role in maintaining the integrity of the high mountain ecosystem by regulating prey populations and maintaining plant community structure. Therefore, it is necessary to understand the role of the snow leopard and its interaction with prey species. Further, elucidating landscape use and co-occurrence of snow leopard and its prey species can be used to assess the differential use of habitat, allowing them to coexist. We used camera trapping and sign survey to study the interactions of snow leopard and its prey species (Siberian Ibex- *Capra sibrica* and Blue sheep-*Pseudois nayaur*) in the Spiti valley Himachal Pradesh. Using the occupancy modelling, we examined whether these prey and predator species occur together more or less frequently than would be expected by chance. To understand this, we have used ten covariates considering the ecology of the studied species. Our results suggest habitat covariates, such as LULC16 (barren area), LULC10 (grassland), ASP (aspect), SLP (slope) and DW (distance to water), are important drivers of habitat use for the snow leopard as well as its prey species. Furthermore, we found that the snow leopard detection probability was high if the site was used by its prey species, i.e., ibex and blue sheep. Whereas, in the case of the prey species, the probability of detection was low when the predator (snow leopard) was present and detected. Besides this, our results suggested that both species were less likely to detect together than expected if they were independent (Snow leopard—Ibex, Delta = 0.29, and snow leopard—blue sheep, Delta = 0.28, both the values are <1, i.e., avoidance). Moreover, despite the predation pressure, the differential anti-predation habitat selection and restriction of temporal activities by the prey species when snow leopard is present allows them to co-exist. Therefore, considering the strong link between the habitat use by the snow leopard and its prey species, it is imperative to generate quantitative long-term data on predator-prey densities and the population dynamics of its prey species in the landscape.

## Introduction

To develop an optimized conservation and management plan for any threatened species, robust information on distribution is imperative [1]. Further, our ability to model and predict the fate of endangered species depends upon recognizing the relative strength and interactions among several population-regulating factors that operate in the ecosystem [2, 3]. The snow leopards are one of the threatened species largely distributed in the high Asian mountains from Southern Siberia in the north to the Himalayan landscape in the south [4, 5]. They have a vast but fragmented distribution across the mountainous landscape of central Asia, and their survival depends primarily on wild ungulates, whose key habitats are alpine regions above the tree line [6, 7]. The snow leopard has been classified as vulnerable by the International Union for Conservation of Nature (IUCN) Red list [8] and it has been provided the highest protection as Schedule-I species of the Indian Wildlife (Protection) Act, 1972 (IWPA). This charismatic species is largely threatened because of the loss of natural prey species, retaliatory killing due to conflict with humans and illegal trade of its fur and bones [8]. Its natural prey base faces competition with domestic livestock in their habitat because of unallocated, and high grazing pressure. The conservation of key prey species is crucial for the survival of any large predator, as changes in preferred prey abundance could alter its population status [9]. As a wide-ranging apex predator, the snow leopard plays a pivotal role in maintaining the ecosystem’s health by maintaining biological integrity and is thus considered an indicator of healthy mountains [10]. The manner in which terrestrial ecosystems are regulated is controversial, but it is undeniable that predators regulate prey populations and, as a result, alter plant community structure [10, 11]. Higher up the mountains, top predators like snow leopards regulate the populations of herbivores such as the blue sheep and Siberian ibex, thereby safeguarding the health of grasslands [11, 12]. A long-term absence of snow leopards could cause trophic cascades as ungulate populations would likely increase, leading to depletion of vegetation cover [12]. Consequently, the loss of this keystone species can lead to regime shifts, alternative ecosystems, and possible losses of ecosystem services which ultimately imbalances the whole ecosystem [10]. Given the role of top predators like snow leopards on ecosystem functioning, snow leopards have a high potential for maintaining ecological integrity [10–12]. Therefore, protecting snow leopards may result in a cascade of benefits to the ecosystem as a whole [13]. Studies suggest that India has relatively rich natural history records of snow leopards which covers different parts of the Himalayas such as Ladakh [7, 14–17], Himachal Pradesh [16, 18–22], Uttarakhand [23, 24], and Sikkim [25]. The studies have covered different aspects of ecology, which have also added to understanding the ecology of the snow leopard & its prey species and interactions between people and wildlife [14, 24]. But the information available on this species is largely focused on Protected Areas (PA) which are considered the best habitats of the species. Maintenance of areas having potential habitat for top predators in and outside the PA’s can serve as a useful tool for conservation and management planning [26]. Due to their elusiveness, non-invasive survey techniques combined with occupancy modelling generate important new insights into the carnivore ecology [27, 28]. The efficiency of camera trapping has been widely used to study snow leopard’s habitat affinities and density estimates [7, 29, 30]. Recent studies show the potential of site occupancy modelling to study patterns in their distribution [30, 31]. We used camera trapping and sign survey to assess the usefulness of this approach and evaluated the co-occurrence patterns of snow leopards and its prey species (Siberian ibex and blue sheep) by using an occupancy model, both two species occupancy model that explicitly assesses co-occurrence and single-species occupancy model considering the imperfect detection [32–35]. We hypothesize that landscape characteristics mainly drive the habitat use of snow leopards and its prey species; we would expect topographic and habitat variables to be the main predictors of occurrence, given the importance of these variables to each species [18, 30, 31]. Furthermore, we also hypothesize that the habitat use of snow leopard and its prey species is mainly driven by predator-prey interaction. If prey availability is a major determinant of the habitat use of snow leopards, we would expect the occupancy and detection probability of snow leopards to be higher when prey species is present and detected. Whereas, if predation is the major determinant of the habitat use of prey species (blue sheep and ibex), we would expect the occupancy or detection probability of prey species to be lower when snow leopard is present or detected *i*.*e*. co-occurrence should be less than expected by chance, predicting avoidance of prey species to predation, considering snow leopard visits frequently those sites where its prey species are present [31, 36]. The knowledge about the relationships among the species will be useful for developing better conservation and management strategies for the long-term viability of snow leopard and its prey species in the landscape of Spiti valley.

## Materials and methods

### Ethical statement

For conducting the research, the research permission was issued by the Principal Chief Conservator of Forest and Chief Wildlife Warden, Government of Himachal Pradesh, with letter no. WL/Research Study/WLM/2291 dated 23/07/2018. Although no animal handling is required in the present study, we have used only camera trap data that is non-invasively collected. Hence, ethical clearance is not required for the present study.

### Study area

The Spiti Valley, with a total area of 7280 km^2^ (31°35’ to 33°0’ N and 77°37’ to 78°35’ E) is a hyper-arid cold desert area with altitudes ranging from 3350 m to 6700 m [37] in the Indian Trans-Himalayas of the Himachal Pradesh State of India [38]. The region is characterized by extreme cold and xeric conditions, with low plant productivity in most parts of the landscape. The temperature ranges from -40° C in winters to 30° C in summer. Being a rain shadow region, the landscape is dry, and the precipitation happens in the form of snow in winter. The vegetation is dominated by dry alpine steppe with gentle-rolling uplands interspersed with steep cliffs and rocky outcrops [39]. Administratively the entire valley is divided into four wildlife ranges, *i*.*e*., i) Pin valley wildlife range, ii) Kibber wildlife range, iii) Tabo wildlife range, & iv) Spiti wildlife range. The valley also has two protected areas, i.e., Pin Valley National Park and Kibber Wildlife Sanctuary, are home to unique and rare species. The most important are snow leopard, blue sheep, ibex, snow cock, red fox and brown bear, which are relatively good in abundance.

### Data collection

**Fig 1:**
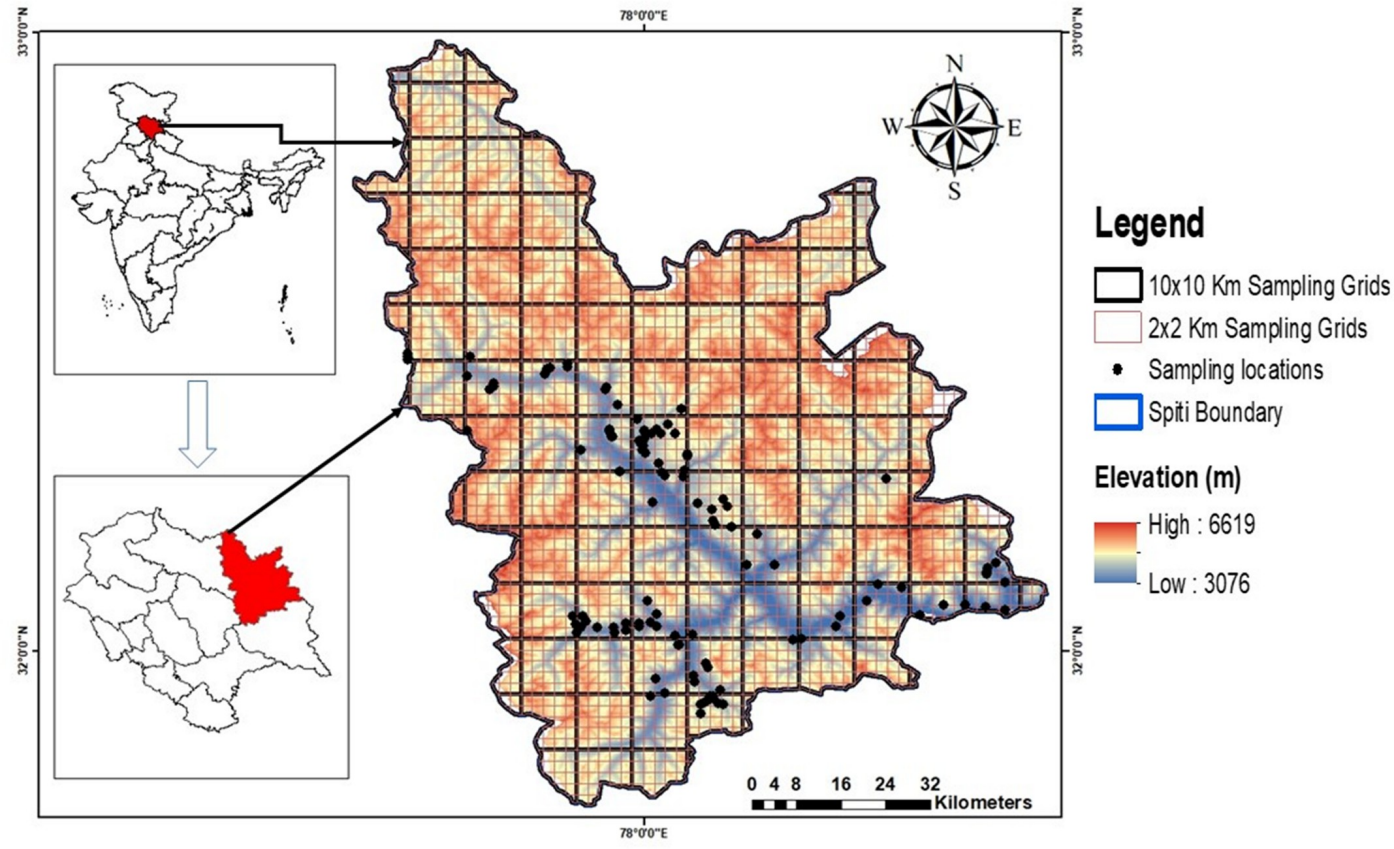
Map of study area Spiti Valley, Himachal Pradesh.

To collect information on the predator-prey relationship, we first stratified the study landscape into different types of LULC (landuse landcover classes) using satellite imagery downloaded from MODIS (Moderate Resolution Imaging Spectroradiometer) and then later divided it into 10×10 Km grids to maximize our sampling effort from July 2018 to August 2019. We have dropped human-dominated areas and grids that were physiologically unsuitable for the species, which is logistically impossible for us to sample. After dropping out of the grids, which were not possible to sample, 29 grids (10×10 km) were selected in which a two-pronged approach, i.e. transect surveys and camera trapping, was used to survey the landscape (Fig 1). Firstly, a reconnaissance survey was conducted in all the selected grids to record species’ presence and absence. All direct or indirect signs of the species were recorded, and grids that possess species signs or sightings were selected for intensive sampling. These intensive sampling grids were further divided into 89 grids of 2×2 km size based on the movement ecology of the study species [40]. Grids were systematically surveyed using an average 2–4 km transects in each grid covering about 440 km. We deployed 117 camera traps on natural trails, vantage points, valleys and near water sources representing all elevation gradients of 3000m to 5051m. We placed ultra-compact SPYPOINT FORCE-11D trail camera (SPYPOINT, GG Telecom, Canada, QC) and Browning Trail Cameras 20 cm above the ground with a minimum distance of approx. 50 m from trails. Two continuous camera-trapping replicate surveys of 30 days each were conducted at each site during the study period. Considering the fact that ibex and blue sheep are group dwelling animals, thus their detection was in groups. Therefore, we consider the single detection for multiple individuals observed in a camera/direct sighting for the analysis from each location. For habitat characterization of the species, we laid 10m radius plots around each camera location & also at all locations where the species was directly (direct sighting) or indirectly (faecal samples) recorded. Information such as GPS location, elevation, aspect, slope, and distance to nearest water source and village were recorded for all sites.

#### Covariates

To understand the influence of habitat variables on the occupancy and detection probability of snow leopard and its major prey species, we used 10 site covariates which were extracted using ArcGIS v. 10.6 software (ESRI, Redlands, CA) from the spatial dataset. These covariates were categorized into three types, i.e., Topographic, LULC (Land use land cover classes) and anthropogenic variables (S1 Table). The topographic variables (elevation, slope and aspect) were generated using a 30x resolution SRTM image downloaded from Earth Explorer (**https://earthexplorer.usgs.gov/**). The LULC classes were identified from classified land use land cover imagery downloaded from MODIS (Moderate Resolution Imaging Spectroradiometer) Land Cover Type Product (MCD12Q1) with a 500-meter resolution. Since our study area falls in a Trans-Himalayan region, we were able to retain the landscape into three broad LULC classes *viz*., Grasslands (LULC 10) dominated by herbaceous annuals, Permanent snow and ice (LULC 15) in which at least 60% of the area is covered by snow for at least ten months of a year, and Barren (LULC 16) at least 60% of the area is non-vegetated barren (sand, rocks, soil) areas with less than 10% vegetation cover. Since much of the landscape was covered by snow and rocky bolder, we have used only three LULC cover types for further analysis considering their importance to species ecology and behaviour. The anthropogenic variable includes distance variables such as distance from the village, distance from a road, distance from the water body, and the Human influence index (Human footprints). The distance values were recorded during the surveys, and the human influence index was downloaded from Socioeconomic Data and Applications Centre SEDAC, NASA (**https://sedac.ciesin.columbia.edu**) [41]. Finally, the values for all the covariates were extracted at 30m spatial resolution, and for generating the point values, we averaged all the pixel values within each sampling site. The Pearson correlation coefficient (rs) was performed to test collinearity among the variables, which indicated that all ten variables were spatially independent (rs<0.7) [42]. Hence, we assumed that each of the covariates could have an ecological effect on the species and therefore used it for further analysis.

### Data analysis

#### Single species occupancy

We used the occupancy modelling framework [32] with a likelihood-based approach to estimate the site occupancy (ψ) of snow leopard and its main potential prey (ibex and blue sheep) and the detection probability of the species (p). The decision to adopt single-season occupancy analysis instead of multi-season was due to heavy coverage of snow throughout the landscape, and the data was collected in a single season only. The detection/non-detection histories for each grid were prepared from camera trap surveys as well as from sign surveys. Those surveys were divided into two sampling occasions of around 30 days each. We pooled the data from all the locations at the respective sites and constructed standard detection histories for each site [43]. In the present study, we adopted a single-season occupancy analysis for estimating the site occupancy probability (ψ) and detection probability (ρ) of the species using a likelihood-based method described by [32]. To avoid numerical optimization of the likelihood [44], all numerical variables were z-standardized, and covariates were dummy-coded. A logit link function [43] was used to model the presence of each species as dependent on habitat covariates in program PRESENCE version 4.0 [43]. Modelling followed a two-step procedure in which a two-stage logistic regression analysis was performed to determine the effect of different site covariates on the occupancy of snow leopard and its prey species. We selected the habitat variables that have an ecological effect on each species. Therefore, several models were developed, including each variable modelled separately and in combination. A total of 38 models were developed for each species, we kept occupancy constant ψ(.) or make it to fluctuate as a function of either the single site covariate or with the combination of two or multiple site covariates [42]. Hence, models were built using the covariates for a plausible biological explanation pertaining to the effects on all study species occupancy (ψ).

Similarly, we developed models including occupancy as constant ψ(.) and variable function of the detection p(covariate) for the respective site covariates. All the candidate models were ranked from highest to lowest based on their AIC (Akaike Information Criterion) values and the Akaike weights [45]. The difference in AIC (∆AIC) between each model and the top model was calculated, which was the basis for inferring each model’s plausibility [46]. The Akaike weight represents the ratio of ∆AIC values for the whole set of candidate models, providing a strength of evidence for each model. Based on the AIC values, only the top-ranking candidate model for each species was used to determine the occupancy in the landscape. The summed model weight of each covariate in these models was used to determine the most influential variables for each species. The sign of logistic coefficient of each variable (positive or negative) was used to determine the direction of influence of the variables on the occupancy and detection probability of each species.

#### Co-occurrence models

The pair-wise species interactions were analyzed using two-species single-season occupancy models [47]. We hypothesized that the presence of a predator influences the occupancy and detection probability of its prey species. These pairs of species included ’snow leopard–ibex’ and ’snow leopard–blue sheep’. To understand the co-occurrence pattern of the predator and its prey species, we have followed the methodology [48]. ψBa/rBa parameterization was implemented within PRESENCE software [43] which investigates pair-wise species interactions assuming that the dominant species was always larger in the analyzed pair [49]. Further, by adopting the strategy suggested by Nagy-Reis et al. [48] for occupancy estimation, the predictors evaluated were: ψA (occupancy of dominant species, Species A), ψBA (occupancy of subordinate species, i.e., species B in the presence of dominant species), and ψBa (occupancy of subordinate species in the absence of the dominant species). Hence, we incorporated the best combination of habitat covariates for ψA, ψBA, and ψBa occupancy models for all the species to investigate the differences in habitat use. Occupancy models were built to evaluate whether the occupancy of dominant species influenced the occupancy of the subordinate species (ψBA≠ψBa) or was independent of the dominant species (ψBA = ψBa). For detection probability, the formulation estimates taken into consideration were: rA (probability of dominant species being detected in the presence of subordinate species), pA (probability of dominant species being detected in the absence of the subordinate species), pB (probability of subordinate species being detected in the absence of dominant species), rBA (probability of detection of subordinate species when the dominant species is present and detected), rBa (probability of subordinate species being detected in the presence of dominant species without detection). Further, we did comparative modelling where we hypothesized that the detection probability of the subordinate species gets influenced by the presence (pB≠rBa; pB≠rBA) or detection (rBa≠rBA) of the dominant species or was independent of the dominant species (pB = rBa = rBA). Further, we modelled with an assumption that the detection of the dominant species can be influenced by the detection of the subordinate species (rA≠pA) or otherwise independent (rA = pA). This strategy was adopted from the study by Nagy-Reis et al. [48] where the co-occurrence Patterns of Neotropical cats were assessed. Since we aimed to investigate species co-occurrence, we compared how likely two species occur together when they are independent of each other. Hence species interaction factor (SIF) for occupancy (phi; [49] and detection probability (delta = rA *rBA /rA*((rA *rBA) + ((1-rA)*rBa)); was calculated following [48, 49]. If two species are present and are detected independently, SIF = 1 (i.e., neutral). If SIF<1, there is species co-occurrence, but the species are detected less frequently than expected if they were independent (i.e., avoidance). If SIF>1, species co-occur but are detected more frequently than expected if independent (i.e., overlap) [49]. A similar procedure was followed as in the case of the single-species analysis for ranking the candidate models. To draw inferences about the co-occurence patterns of snow leopard and its prey species, the top-ranked model with minimum AIC value, the estimated parameters (ψA, ψBA, ψBa, rA, pA, pB, rBa, rBA) and the SIF calculated for each species pairs were taken into consideration [48].

#### Activity pattern

The daily activity pattern of all the species and the overlap between the temporal patterns of species sharing the same habitat were also estimated. We have compared the activity pattern of each species to understand the overlapping patterns using package "Overlap" in R (R Development Core Team) and assess how the snow leopard activity is influencing the daily activity pattern of its prey. The time and date printed on the photographs have been used to determine the daily activity pattern of individual species [50]. We used a Daily Activity Index (DAI) of an hour duration to examine the daily activity [51]. The coefficient of overlap is denoted by "Dhat1" values, ranging between zero (no overlap) and 1.0 (complete overlap).

## Results

### Single species occupancy modelling

**Fig 2:**
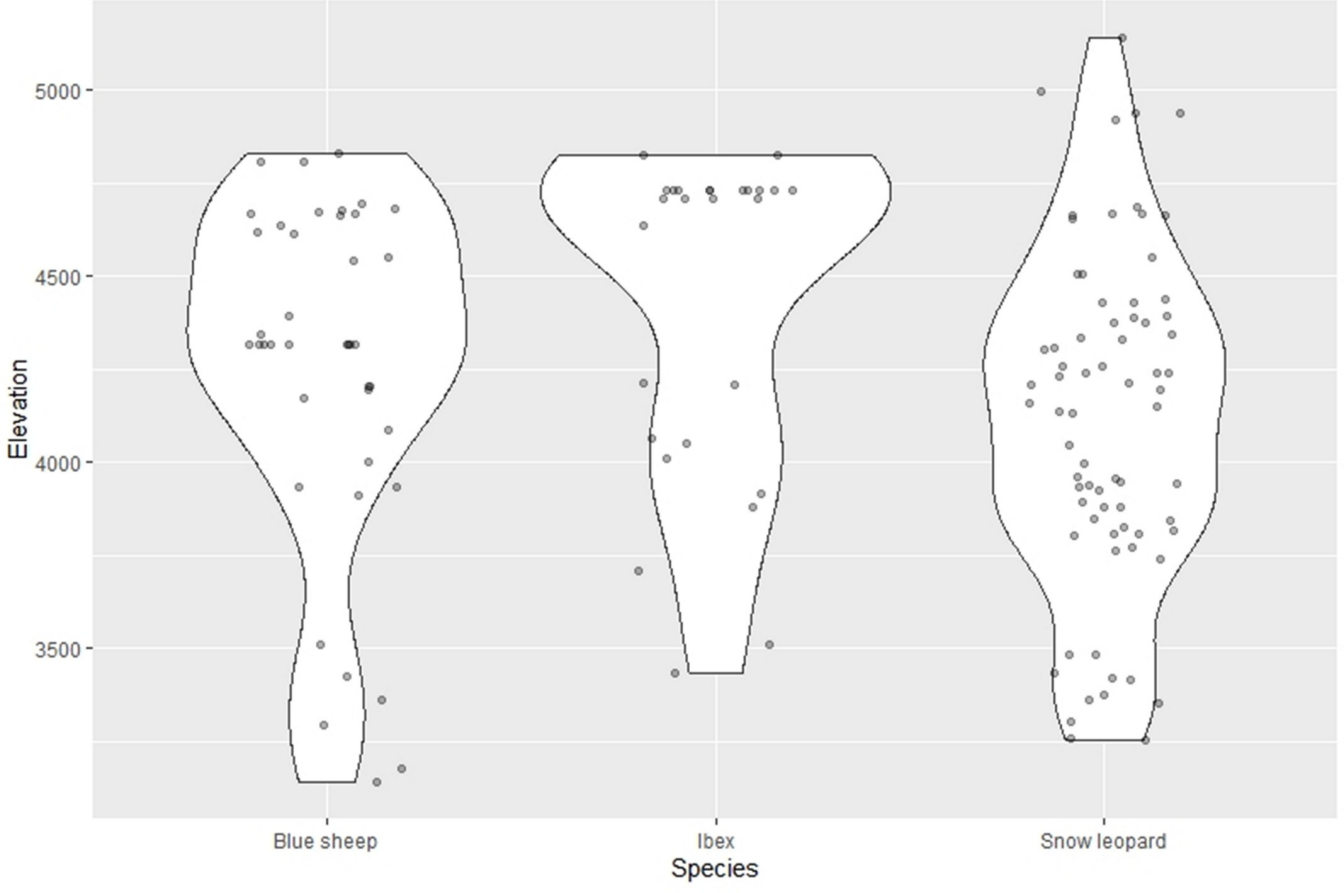
Violin plot displaying the elevational distribution over which snow leopard and its prey species were detected.

The total sampling effort of 3152 camera trap nights yielded 93 independent detections consisting of 45 independent detections of snow leopard, 23 detections (considered single detection for group individuals captured) of ibex, and 25 of blue sheep in Spiti valley. Twenty-seven signs of ibex, 85 signs of snow leopard and 36 signs of blue sheep, accounting for a total of 148 signs, were recorded by traversing 54 trails of 440 km at different locations. Altitudinal range overlap between snow leopard (3251–5140 m) and its prey species (Ibex = 3432–4828 m, blue sheep = 3141–4807 m) is quite prominent (Fig 2). We had evidences that the habitat use of each of the species was influenced by habitat covariates.

### Snow leopard (predator)

The highest captures of snow leopard were in the Kibber Wildlife range (n = 26) followed by Pin valley national park (n = 16) and (n = 3) in the Tabo Wildlife range. The combination of both, i.e., camera traps and sign survey, yielded 0.28 naïve occupancy of snow leopard in Spiti Valley. It was quite evident that the occupancy and detection probability of snow leopard was influenced by the habitat covariates explained by the top 5 models (S2 Table). The top-ranked model based on the lowest AIC value indicated that occupancy and detection probability of snow leopard was influenced the most by keeping occupancy constant psi(.) and varying detection probability p(LULC16), *i*.*e*., barren area (S2 Table). This model assumed that the detection probability of snow leopard was positively influenced by LULC 16 (β = 0.16±0.02) (S3 Table).

### Prey species of snow leopard (Ibex and Blue sheep)

**Fig 3:**
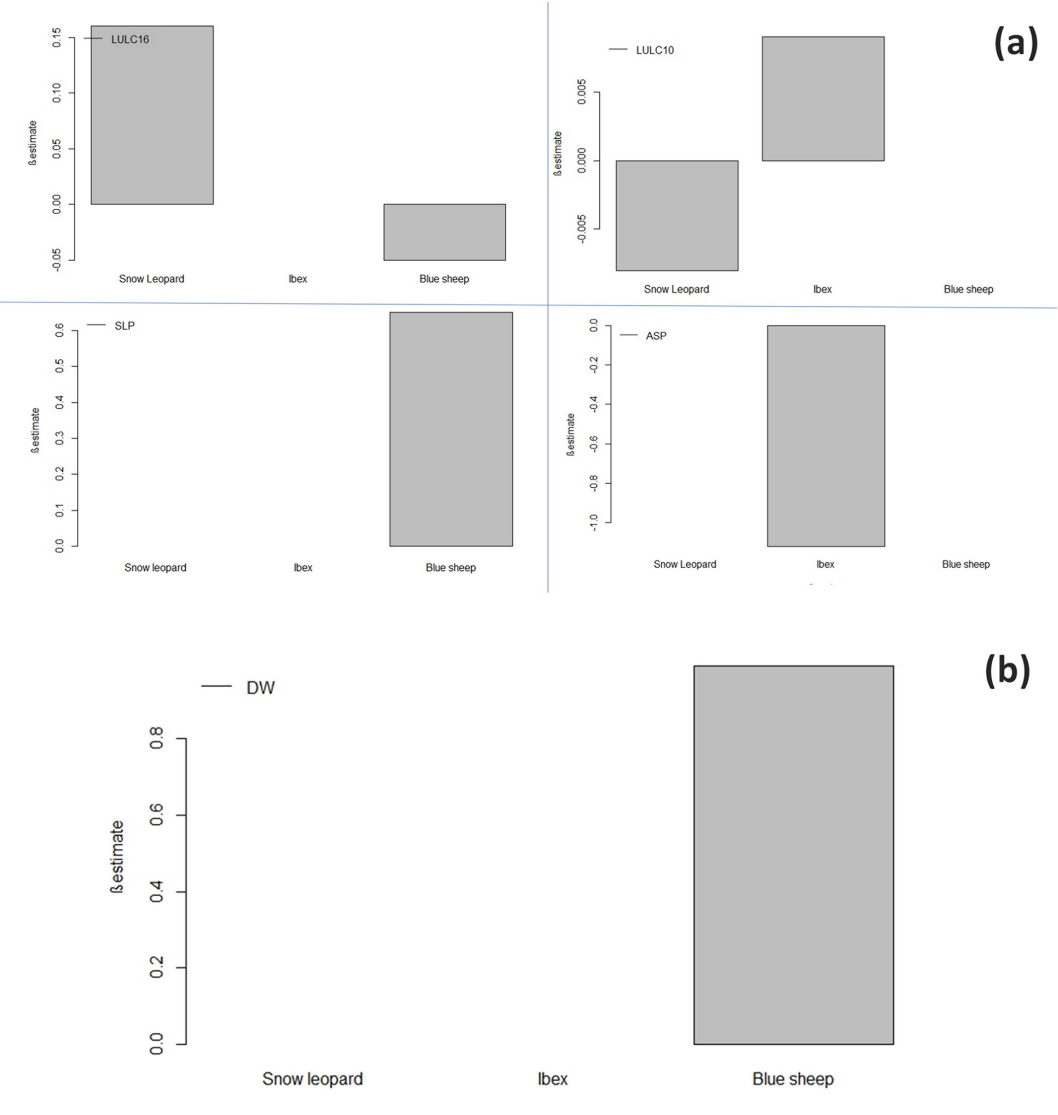
**a and b.** Influence of habitat covariates retained in top models on the occupancy of snow leopard and its prey species. Variables retained are LULC16- barren area, LULC10- grasslands/herbaceous annuals, ASP-aspect, SLP-slope &DW-distance to water.

Ibex had the highest captures in Pin Valley National Park (n = 15), followed by the Kibber Wildlife range. On the counterpart, blue sheep had the highest captures in the Kibber Wildlife range (n = 16), followed by the Tabo Wildlife range (n = 10). The combination of both, *i*.*e*., camera traps and sign survey, yielded naïve occupancy of ibex (0.21) and blue sheep (0.19) in Spiti Valley. Results of the present study inferred that aspect and LULC 10 were the main factors influencing the occupancy and detection probability of ibex (Fig 3A, S2 Table). The top-ranked model with lowest AIC suggests that the occupancy of ibex varied as a function of (ASP and LULC 10) and detection probability varied as a function of (LULC10) (Table 1). We found that variable ASP (β = -1.12±0.48) had a negative influence on the occupancy of ibex, while on the contrary, grasslands had a positive influence on the occupancy (β = 0.0009±0.0008) and detection probability (β = 0.15±0.03) of ibex (Fig 3A, S3 Table). Similarly, occupancy and detection probability of blue sheep having occupancy as a variable function of (DW- distance to water, SLP- slope, and LULC16 -barren area) and detection probability constant (.) emerged as a top-ranked model. This model suggests that the occupancy probability of blue sheep was positively influenced by variables DW (β = 0.99±0.76), SLP (β = 0.65±0.44) and negatively influenced by variable LULC 16 (β = -0.05±0.06) (Fig 3A & 3B).

**Table 1:** Co-occurrence occupancy models used to evaluate the role of predator-prey interactions on the habitat use of predator and prey in Spiti Valley.

Predator prey Interaction model	AIC	deltaAIC	*wi*	Model Likelihood	no.Par.	-2L
**Snow leopard-Ibex**						
ψ A(LULC16) ≠ ψ BA(LULC10) ≠ ψ Ba (ASP), pA = pB = rA, = rBA = rBa	120.56	0	0.6602	1	8	104.56
ψ A(LULC16) = ψ BA(LULC16) = ψ Ba (LULC16), pA≠pB,rA≠rBA≠rBa	124.45	3.89	0.0944	0.143	8	108.45
ψ A(LULC16) ≠ ψ BA(ASP) = ψ Ba (ASp), pA≠pB≠rA ≠rBA≠rBa	125.15	4.59	0.0665	0.1008	8	109.15
ψ A(LULC16) = ψ BA(LULC16) ≠ ψ Ba (ASP), pA≠pB≠rA≠rBA≠rBa	125.26	4.7	0.063	0.0954	8	109.26
ψ A(LULC16) = ψ BA (LULC16) = ψ Ba (LULC16), pA≠ pB≠ rA≠ rBA≠ rBa	127.35	6.79	0.0221	0.0335	8	111.35
ψ A ≠ ψ BA = ψ Ba, pA, = pB = rA, = rBA, = rBa	129.63	9.07	0.0071	0.0107	6	117.63
ψ A(LULC16) = ψ BA(LU16) = ψ Ba (LU16), pA ≠ rA ≠ pB = rBA = rBa	131.72	11.16	0.0025	0.0038	8	115.72
**Snow leopard-blue sheep**						
ψ A(SLP) ≠ ψ BA(LULC10) ≠ ψ Ba (Dw), pA = pB≠rA, ≠rBA≠rBa	127.89	0	0.281	1	8	111.89
ψ A(LULC16) ≠ ψ BA(SLP) ≠ψ Ba (DW), pA = pB≠rA≠rBA≠rBa	127.91	0.02	0.2782	0.99	8	111.91
ψ A(LULC16) ≠ψ BA(LULC10) ≠ ψ Ba (DW), pA = pB = rA = rBA =, rBa	129.74	1.85	0.1114	0.3965	8	113.74
ψ A = ψ BA = ψ Ba, pA≠pB≠rA≠rBA≠rBa	129.77	1.88	0.1098	0.3906	8	113.77
ψ A(LULC16) = ψ BA(LULC10) ≠ ψ Ba (DW), pA = pB,rA≠rBA≠rBa	130.65	2.76	0.0707	0.2516	8	114.65
ψ A(SLP) ≠ψ BA(LULC10) ≠ ψ Ba (DW), pA = pB,rA = rBA = rBa	132.53	4.64	0.0276	0.0983	8	116.53

Note: ψA = occupancy of dominant species; ψBA = occupancy of subordinate species in the presence of dominant species; ψBa = occupancy of subordinate species in the absence of the dominant species on the site. rA (detection probability of dominant species detection in the presence of subordinate), pA (detection probability of the dominant species in the absence of subordinate species), pB (detection probability of subordinate species in the absence of dominant species), rBA (detection probability of subordinate species in the presence and detection of dominant species), rBa (detection probability of subordinate in the presence and without detection of dominant species).We used " = " to designate that two or more parameters were set as equal (e.g., ψBA = ψBa means that the occupancy of the subordinate species is independent of that of the dominant species).

### Predator-prey interaction (co-occurrence)

**Fig 4:**
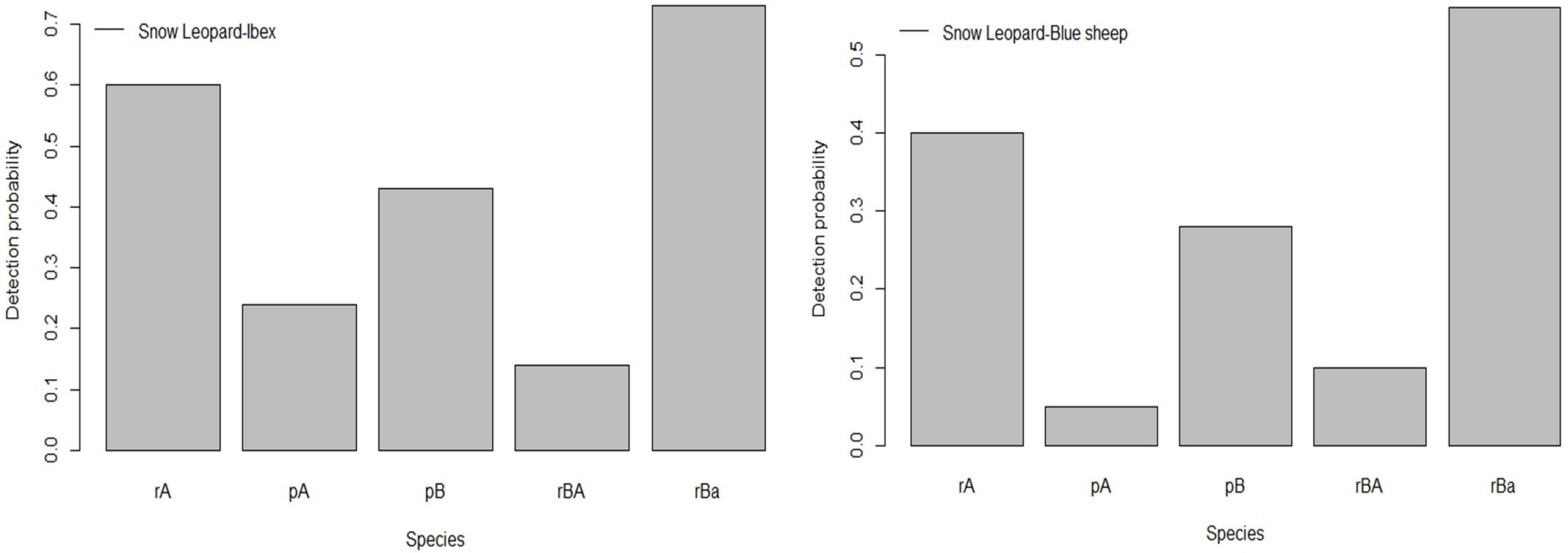
Influence of snow leopard on detection of its prey species, where: rA (probability of dominant species detection in the presence of subordinate), pA (detection probability of the dominant species in the absence of subordinate species), pB (detection probability of subordinate species in the absence of dominant species), rBA (detection probability of subordinate species in the presence and detection of dominant species), rBa (detection probability of subordinate in the presence and without detection of dominant species).

The best-supported model estimated the probability of occupancy of the ’snow leopard–ibex’ and ’snow leopard-blue sheep’, but we did not find any evidence that occupancy of ibex or blue sheep was influenced by snow leopard (Tables 1 and 2). The species did not favour any of the variables where the pair had similar occupancy, implying that the subordinate species had similar occupancy regardless of whether the dominant species was present on-site or not (i.e., ψA = ψBA = ψBa) (Table 2). However, we found that the presence of prey species, i.e. ibex and blue sheep have an effect on the presence and detection probability of predator, the more dominant species, snow leopard (i.e., that pA≠rA), or Vice-versa (i.e., pB≠rBA ≠ rBa or pB≠rBA = rBa (Fig 4, Table 2). Our model established that one species affects the behaviour and landscape utilization of the other since the snow leopard is more likely to be detected, if its prey species, i.e., ibex and blue sheep are present while, its prey species are less likely to be detected when snow leopard is present and detected (Fig 4). Moreover, the detection probability of prey species was five times higher when the snow leopard was present but not detected, than when the snow leopard was present and detected (Table 2). Furthermore, results also suggested that both the species were less likely to detect together than would be expected if they were independent (Snow leopard—Ibex, Delta = 0.29, and snow leopard—blue sheep, Delta = 0.28, both the values are <1, i.e., avoidance) (Table 2).

**Table 2:** Occupancy (ψ), detection probability (p and r), and species interaction factor (SIF—phi and Delta) estimated from co-occurrence occupancy models of predator prey interaction in Spiti Valley.

Interaction	ΨA	ΨBA	ΨBa	rA	pA	pB	rBA	rBa	Phi	Delta
**Snow leopard-Ibex**	0.35	0.35	0.35	0.60	0.24	0.43	0.14	0.73	1.00	0.29
**Snow leopard-blue sheep**	0.62	0.62	0.62	0.40	0.05	0.28	0.10	0.56	1.00	0.28

Note: ψA = occupancy of dominant species; ψBA = occupancy of subordinate species in the presence of dominant species; ψBa = occupancy of subordinate species in the absence of the dominant species on the site. rA (detection probability of dominant species detection in the presence of subordinate), pA (detection probability of the dominant species in the absence of subordinate species), pB (detection probability of subordinate species in the absence of dominant species), rBA (detection probability of subordinate species in the presence and detection of dominant species), rBa (detection probability of subordinate in the presence and without detection of dominant species). Phi = ratio of how much more (>1) or less (1) or less (<1) likely the species are to co-occur at a site compared to what would be expected if the species occurred independently of each other; Delta = ratio of how much more (>1) or less (<1) likely the species are to be detected together compared to what would be expected if they were detected independently.

### Activity pattern

**Fig 5:**
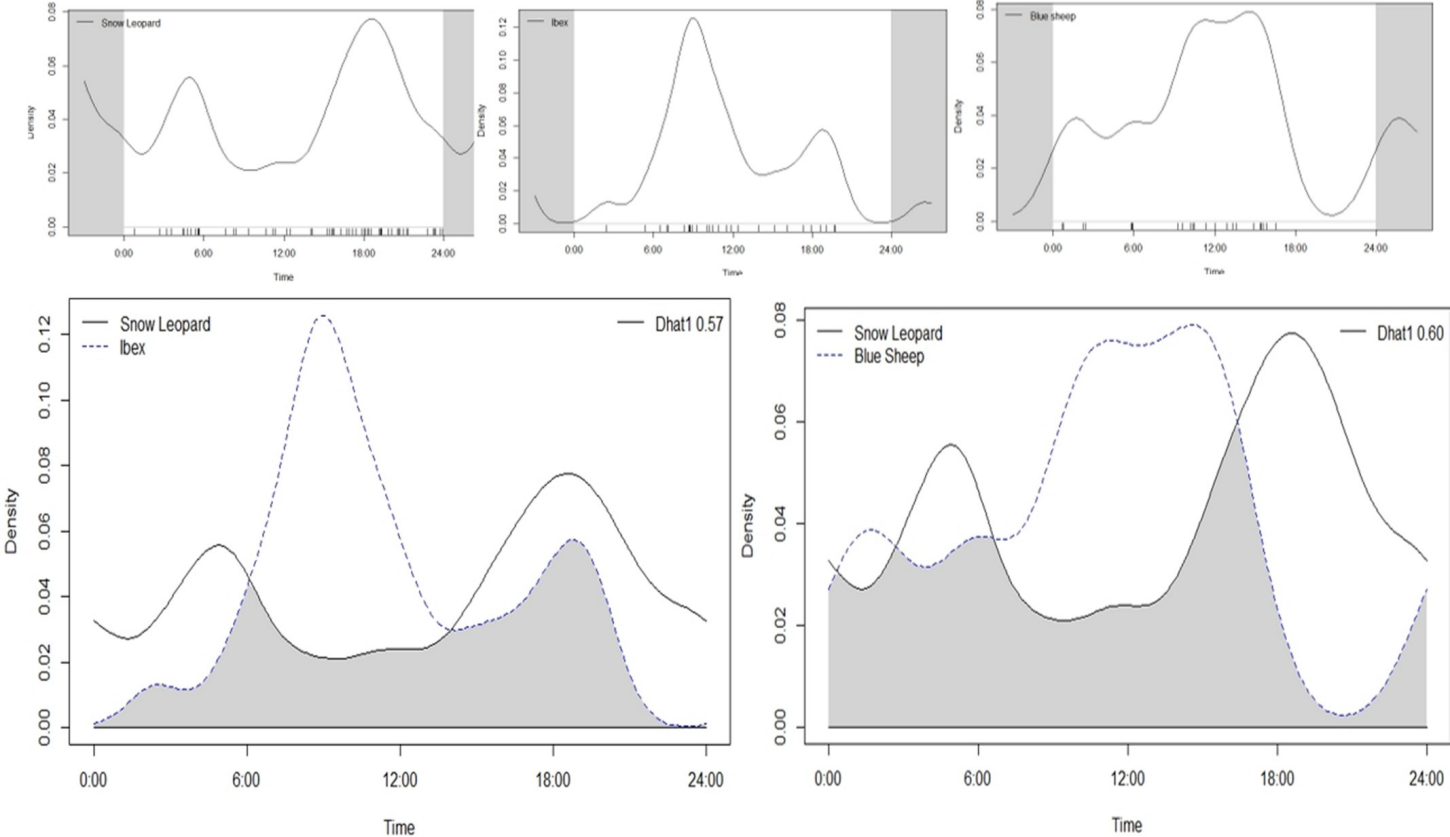
Temporal activity pattern of Snow leopard, Ibex and Blue sheep (top row) and overlapping activity pattern of predator-prey (bottom row) in Spiti Valley.

On the visualization, the daily activity pattern of snow leopard and its prey species depicts the marked difference of their activity peaks (Fig 5). The snow leopard is mostly active at dawn and showed greater peak activities in the late evening hours till morning 6.00 hrs, and it gradually decreased towards mid-day. On the contrary, the ibex showed its peak activity when snow leopard is least active from 6.00hrs to 12.00 hrs and gave a smaller peak before sunset (Fig 5). However, blue sheep was most active throughout the day between 09:00 to 18:30 hrs and showed slight activity in early morning hrs. The overlap plot of temporal activity between the predator and its prey species, i.e., ibex and blue sheep, showed a marked difference in their activity due to the presence of snow leopard (Snow leopard–Ibex Dhat1 = 0.59 & Snow leopard–blue sheep Dhat1 = 0.60) (Fig 5).

## Discussion

The predator-prey interaction is one of the key topics of behavioural ecology because predation significantly influences prey population dynamics and behaviour and the ecosystem functioning [11, 16, 52]. In the present study, we used occupancy modelling [32] for incorporating the detection probability with the habitat variables whilst exploring the co-occurrence patterns and site utilization by the snow leopard and its prey species. Snow leopards’ position as a flagship species for their ecosystem has given them an ambassadorial role. However, the species is listed as vulnerable by IUCN red list [53] and Schedule 1 species in IWPA. Yet, its future is uncertain due to the vulnerability of ever ending anthropogenic activities [7, 54, 55] and climate change to their population and habitats [56]. Therefore, when investigating species habitat use, it is crucial to identify conservation areas and assess the relative importance of habitat variables and interaction with other co-occurring species that govern species’ distribution and habitat use. Our first hypothesis that the topographic and habitat variables would be an important predictor of landscape use by snow leopard and its prey species is corroborated with the previous findings [7, 15, 18, 30, 31]. We demonstrated that the variable LULC16 (barren area) is the main factor influencing the habitat use of snow leopards with a naïve occupancy of 0.28 in the Spiti valley. This model assumed that snow leopards had higher detection probability at barren or non-vegetated areas (LULC16), keeping occupancy probability as constant psi(.) wherein (LULC 16) positively (β = 0.16±0.02) influenced the detection probability of snow leopard (**S1 Table**). Our results were in concordance with other studies [14, 15, 57, 58], which suggest snow leopards mostly occur in the more arid, nonforested or barren areas in India between 3200 m and 5200 m.

The high altitudes of the Indian Himalayas support a diverse and unique assemblage of wild flora and fauna [59, 60], such as Siberian ibex (*Capra sibirica*) and bharal (*Pseudois nayaur*), which constitute the primary prey of snow leopards [59, 61]. Both the prey species of snow leopard are considered as Least concern in IUCN red list [53]. Occupancy modelling yielded a naïve occupancy of (0.21) (Ibex) and (0.19) blue sheep in Spiti Valley. We demonstrated that ASP (i.e. aspect) and LULC10 (i.e. Grasslands—dominated by herbaceous annuals), as a variable function of occupancy with high relative importance, were the main factors influencing their habitat use ibex. We found that occupancy and detection probability of ibex was negatively influenced by aspect (ASP) (β = -1.12±0.48) and positively influenced by grassland (LULC10) (β = 0.0009±0.0008) (**S3 Table**). Further, our model results are in coherence with the findings of Bhatnagar (1997) [18] and Newmark and Rickart (2012) [62] which suggest a negative association of ibex with the north-facing aspect because ibex primarily chooses southerly aspects (SE, S and SW), especially the south-facing slopes, which are used more during all seasons. The reason that southern slopes also are the first areas where sprouting initiates in late winter/spring provides more justification for the use of southern aspects by ibex. As the snow melts from spring to summer, more areas become conducive for ibex and can move into other areas. Our findings were consistent with Bhatnagar (1997) [18], which suggests that ibex occupies grasslands or plant cover areas used more in summer and autumn with clear avoidance of barren areas with high rockiness.

Similarly, we demonstrated occupancy and detection probability of blue sheep having (DW- distance to water, SLP- slope, and LULC16 -barren area) as a variable function of occupancy and kept detection probability as constant p(.) (**S2 Table**). This model explains that the occupancy probability of blue sheep was positively influenced by variables DW (β = 0.99±0.76), SLP (β = 0.65±0.44) and negatively influenced by variable LULC 16 (β = -0.05±0.06), which had a positive association with snow leopard (**S3 Table**). Our hypothesis is endorsed by the previous studies, which suggest water sources with open vegetation are the main habitat for drinking and foraging for the blue sheep, while it uses steep slopes in the mountains for resting and bedding [63, 64].

Furthermore, since species present at a site are not always detected with certainty, incorporating the detection probability with the habitat selection directly into the model may evade incorrect inferences about co-occurrence patterns as suggested by MacKenzie et al. 2004 [47]. We adopted this approach and found no evidence that the snow leopard occupancy negatively influences the occupancy of its prey species. However, the best model estimated the probability of occupancy of the ’snow leopard–ibex’ and ’snow leopard-–blue sheep’, but we did not find any evidence that occupancy of ibex or blue sheep was influenced by snow leopard (Tables 1 and ***2***). The species did not favour any of the variables where the pair had similar occupancy, implying that the subordinate species had similar occupancy regardless of whether the dominant species was present at the sampling site or not (i.e., ψA = ψBA = ψBa) (Table ***2***). However, we found that the presence of prey species, *i*.*e*. ibex and blue sheep have a positive effect on the presence and detection probability of predator, the more dominant species, snow leopard (i.e., that pA≠rA), or Vice-versa (i.e., pB≠rBA ≠ rBa or pB≠rBA = rBa (Table ***2***). Furthermore, results also suggested that both the pair of species were less likely to detect together than would be expected if they were independent (delta <1 avoidance; Table ***2***). We found that one species affects the behaviour and site utilization pattern of the other as Snow leopard was more likely to be detected if its prey species, *i*.*e*. ibex and blue sheep were present while its prey species were less likely to be detected when snow leopard was present and detected.

Moreover, the detection probability of prey species was five times higher when the snow leopard was present but not detected than when the snow leopard was present and detected (Table ***2***). Our results corroborated with the previous studies [7, 31, 36], which suggests that there is a positive association between prey presence and site use by a snow leopard. However, the snow leopards appear to visit sites with greater prey presence [30]. Our results were consistent with previous findings [18, 65], which suggested substantial predation pressure in the landscape. Prey species may be at lower risk of predation by selecting habitats that serve as an effective refuge against predators. The difference in the use of habitats is clearly shown by the positive association of blue sheep with slopes and negative association with barren areas, which had a positive association with snow leopard. Likewise, ibex uses aspect and LULC10 the most, with the least support for the snow leopard, which can be attributed to species-specific anti-predatory behaviour [66]. Our results suggest that the prey species probably needs to strike a balance between habitat use and predator avoidance, which is evident by our results (Table ***2***). Moreover, the overlapping plot of temporal activity between the predator and its prey species, *i*.*e*. ibex and blue sheep, showed a marked difference in their activity due to the presence of snow leopard (Snow leopard–Ibex Dhat1 = 0.59 & Snow leopard–blue sheep Dhat1 = 0.60) (Fig 5). The snow leopard is mostly active at dawn and shows greater activities in the late evening hours. However, ibex and blue sheep showed their peak activity when the snow leopard was least active (Fig 5). The study has shown that the snow leopard and its prey species coexist in Spiti valley despite the predation pressure, apparently by differential anti-predator habitat selection and restriction of temporal activities by the prey species when the snow leopard is present. The differential use of habitat by the prey species suggests presumably a reduced possibility of predation or clear avoidance by prey species.

Furthermore, our study is also supported by the landscape of fear hypothesis, which states that the habitat used by prey species comprises high to low-risk patches as determined by the presence and ubiquity of predators within an ecosystem [67, 68]. This might be the reason that the prey species tend to avoid those patches which have a high risk of predation. This results in a landscape of risky versus safe areas for prey species that can be reflected in the quality and availability of habitat [68].

The endangered snow leopard, subsisting in a seemingly isolated and remote landscape, is potentially at risk of anthropogenic disturbances [6, 69, 70]. Snow leopards are threatened by retaliatory killings, livestock depredation, prey declines, disease and illegal trade [6]. In conclusion, our results procured habitat covariates, such as LULC16, LULC10, ASP, SLP, and DW, as important drivers of habitat use for snow leopard and its prey species. The predator-prey interactions are important drivers of snow leopard and its prey species’ habitat use. Our results support a strong link between the habitat use of snow leopard and its prey species, which highlights the need for conservation programmes to include its prey species, despite the fact that these species are classified as least concern by the IUCN. It is evident that despite the predation pressure, the differential anti-predation habitat selection and restriction of temporal activities by the prey species when snow leopard is present allows them to co-exist. We suggest a need to monitor prey populations of snow leopards at the local level and for further research into the ecology of its prey species in the study landscape. Specifically, the quantitative long-term data is lacking on predator-prey densities and the population dynamics of its prey species in the landscape. Attention is needed to avert any risks to snow leopards associated with ever ending development activities, such as the construction of roads, towers and grazing pressure by livestock.

## Supporting information

S1 Table Habitat variables used for landscape use and cooccurrence of snow leopard and its prey species. (DOCX)

S2 Table Single-season single-species top five occupancy models used to evaluate the influence of different variables on the habitat use of snow leopard and its prey species in Spiti Valley. (DOC)

S3 Table β-estimates of single season occupancy models to evaluate influence of covariates on habitat use of snow leopard and its prey species (Ibex & blue sheep) in Spiti Valley. (DOC)
