# Supplementary material for: Landscape use and co-occurrence pattern of snow leopard (Panthera uncia) and its prey species in the fragile ecosystem of Spiti Valley, Himachal Pradesh
Source: PLoS One. 2022 Jul 21;17(7):e0271556. doi: 10.1371/journal.pone.0271556 (PMC9302832; doi:10.1371/journal.pone.0271556)
Supplement: S1 Table — (DOCX) [file pone.0271556.s001.docx]

Supplementary Table 1: Habitat variables used for landscape use and cooccurrence of snow leopard and its prey species.

| **S.No** | **Variable** | **Code** | **Data** | **Source** |
| --- | --- | --- | --- | --- |
| 1 | Grass land (dominated by herbaceous annuals) | LULC 10 | MCD12Q1 | USGS |
| 2 | Permanent snow and ice (at least 60% of area is covered by snow for at least 10 moths of a year) | LULC 15 |  |  |
| 3 | Barren (at least 60% of the area is non vegetated barren (sand, rocks, soil) areas with less than 10% vegetation cover). | LULC 16 |  |  |
| 4 | Distance to water | DW | Calculated using log Euclidean distance (Arcgis 10) | LULC map |
| 5 | Distance to road | DR |  |  |
| 6 | Distance to village | DV |  |  |
| 7 | Elevation | ELE | SRTM | USGS |
| 8 | Slope | SLP |  |  |
| 9 | Aspect | ASP |  |  |
| 10 | Human foot print | HFP | EARTHDATA | SEDAC |
