# Supplementary material for: Landscape use and co-occurrence pattern of snow leopard (Panthera uncia) and its prey species in the fragile ecosystem of Spiti Valley, Himachal Pradesh
Source: PLoS One. 2022 Jul 21;17(7):e0271556. doi: 10.1371/journal.pone.0271556 (PMC9302832; doi:10.1371/journal.pone.0271556)
Supplement: S2 Table — (DOC) [file pone.0271556.s002.doc]

**Landscape use and cooccurrence of Snow leopard and its prey species in the fragile ecosystem of Spiti valley-Supporting Information**

S1 Table: Single-season single-species top five occupancy models used to evaluate the influence of different variables on the habitat use of Snow leopard and its prey species in Spiti valley.

| **Model** | **AIC** | **deltaAIC** | ***Wi*** | **Model Likelihood** | **no.Par.** | **-2L** |
| --- | --- | --- | --- | --- | --- | --- |
| **Snow leopard** | | | | | | |
| ψ (.), p(LULC16) | 114.92 | 0 | 0.3397 | 1 | 2 | 110.92 |
| ψ (.), p(LULC16+DR) | 116.91 | 1.99 | 0.1256 | 0.3697 | 3 | 110.91 |
| ψ (LULC10), p(LULC16) | 117.41 | 2.49 | 0.0978 | 0.2879 | 2 | 113.41 |
| ψ LULC10+LULC16), p(LULC16) | 118.04 | 3.12 | 0.0714 | 0.2101 | 3 | 112.04 |
| ψ (LULC10), p(.) | 121.96 | 7.04 | 0.0101 | 0.0296 | 2 | 117.96 |
| **Siberian Ibex** | | | | | | |
| ψ (ASP+LULC10), p(LULC10) | 159.81 | 0 | 0.4212 | 1 | 3 | 153.81 |
| ψ (ASP), p(LULC10) | 160.59 | 0.78 | 0.2851 | 0.6771 | 2 | 156.59 |
| ψ (ASP+LULC10), p(LULC10+DR) | 160.8 | 0.99 | 0.2567 | 0.6096 | 4 | 152.8 |
| ψ (.), p(LULC10) | 166.5 | 6.69 | 0.0148 | 0.0353 | 2 | 162.5 |
| ψ ASP), p(.) | 168.43 | 8.62 | 0.0057 | 0.0134 | 2 | 164.43 |
| **Blue sheep** | | | | | | |
| ψ (DW+SLP+LULC16), p(.) | 153.57 | 0 | 0.2031 | 1 | 4 | 145.57 |
| ψ (DW+LULC16), p(.) | 154.07 | 0.5 | 0.1581 | 0.7788 | 3 | 148.07 |
| ψ (DW+DR), p(.) | 155.32 | 1.75 | 0.0846 | 0.4169 | 3 | 149.32 |
| ψ (LULC16), p(.) | 156.03 | 2.46 | 0.0594 | 0.2923 | 2 | 152.03 |
| ψ (HFP), p(.) | 156.87 | 3.3 | 0.039 | 0.192 | 2 | 152.87 |

LULC16-barren area, LULC10- grassland, DR- distance to road, ASP-aspect, DR-distance to road, DW-distance to water, SLP-slope, HFP-human footprint
