# Supplementary material for: Landscape use and co-occurrence pattern of snow leopard (Panthera uncia) and its prey species in the fragile ecosystem of Spiti Valley, Himachal Pradesh
Source: PLoS One. 2022 Jul 21;17(7):e0271556. doi: 10.1371/journal.pone.0271556 (PMC9302832; doi:10.1371/journal.pone.0271556)
Supplement: S3 Table — (DOC) [file pone.0271556.s003.doc]

**Landscape use and cooccurrence of Snow leopard and its prey species in the fragile ecosystem of Spiti valley-Supporting Information**

S2 Table: β-estimates of single season occupancy models to evaluate influence of covariates on habitat use of snow leopard and its prey species (Ibex & blue sheep) in Spiti valley.

| **Model** | **Ψ(.)** | **Ψ(LULC16)** | **Ψ(LULC 10)** | **Ψ(DR)** | **Ψ(ASP)** | **Ψ(SLP)** | **Ψ(DW)** | **Ψ(HFP)** | **P(.)** | **P(LULC10)** | **P(LULC 16)** | **P(DR)** |
| --- | --- | --- | --- | --- | --- | --- | --- | --- | --- | --- | --- | --- |
| Ψ (.), p(LULC16) | 25.63±29.88 | 0.16±0.12 | - | - | - | - | **-** | - | - | - | - | - |
| Ψ(.), p(LULC16+DR) | 10.50±18.32 | -0.001±0.005 | - | -0.13±0.02 | - | - | **-** | - | - | - | - | - |
| Ψ (LULC10), p(LULC16) | - | **-** | -0.008±0.008 | - | - | - | **-** | - | - | - | -0.07±0.03 | - |
| Ψ (LULC10+LULC16),  p(LULC16) | - | -0.006±0.008 | -0.008±0.008 | - | - | - | **-** | - | - | - | -0.07±0.03 | - |
| Ψ (LULC10), p(.) | - | - | -0.02±0.08 | - | - | - | **-** | - | -0.54±0.38 | - | - | - |
| Ψ (ASp+LULC10)  ,p(LULC10) | - | - | 0.009±0.008 | - | -1.12±0.48 | - | **-** | - |  | -0.15±0.03 | - | - |
| Ψ (ASp), p(LULC10) | - | - | - | - | -0.95±0.43 | - | **-** | - |  | -0.15±0.03 | - | - |
| Ψ (Asp+LULC10),  p(LULC10+DR) | - | - | -0.008±0.008 | - | -1.16±0.48 | - | **-** | - |  | -0.16±0.03 | - | -0.29±0.29 |
| Ψ (.), p(LULC10) | -0.19±0.42 | - | - | - | - | - | **-** | - |  | -0.14±0.05 | - | - |
| Ψ (ASp), p(.) | - | - | - | - | -0.87±0.43 | - | **-** | - | -0.82±0.24 |  | - | - |
| Ψ (DW+SLP+LULC16)  ,p(.) | - | 0.05±0.06 | - | - | - | 0.65±0.44 | 0.99±0.7 | - | -0.69±0.49 |  | - | - |
| Ψ (DW+LULC16)  ,p(.) |  | -0.001±0.008 | - |  | - | - | **-** | - | -0.93±0.25 | - | - | - |
| Ψ (DW+DR)  ,p(.) |  | - | - | -0.48 ±0.42 | - | - | 1.36±0.77 | - | - | - | - | - |
| Ψ (LULC16), p(.) |  | -0.06±0.04 | - | - | - | - | **-** | - | -0.53±0.48 | - | - | - |
| Ψ (hfp), p(.) | - | - | - | - | - | - | **-** | 0.17±0.20 | -1.25±0.33 | - | - | - |

LULC16-barren area, LULC10- grassland, DR-distance to road, ASP-aspect, DR-distance to road, DW-distance to water, SLP-slope, HFP-human footprint
